# CD8^+^ T cells are increased in the subventricular zone with physiological and pathological aging

**DOI:** 10.1111/acel.13198

**Published:** 2020-08-01

**Authors:** Manuel Moreno‐Valladares, Leire Moreno‐Cugnon, Tulio Mateo Silva, Juan Pablo Garcés, Ander Saenz‐Antoñanzas, María Álvarez‐Satta, Ander Matheu

**Affiliations:** ^1^ Biodonostia Health Research Institute Group of Cellular Oncology San Sebastian Spain; ^2^ Pathology Department Donostia University Hospital San Sebastian Spain; ^3^ CIBER of Frailty and Healthy Aging (CIBERfes) Carlos III Institute Madrid Spain; ^4^ IKERBASQUE Basque Foundation for Science Bilbao Spain

**Keywords:** Aging, CD8^+^ cells, dentate gyrus, neurodegenerative diseases, subventricular zone, T‐cell infiltration

## Abstract

Age‐related cognitive decline and neurodegenerative diseases are associated with less functional neurogenic niches. It has been recently shown that aged subventricular zone (SVZ) suffers an infiltration of T cells, which affects neural stem cell activity in mice. Whether this occurs in human neurogenic niches or to which extent T‐cell infiltration is also taking place in neurodegenerative diseases remains unknown. In this work, we studied the presence of T cells in both human neurogenic niches in young and old individuals. There was a significant increase in the number of CD3^+^ and CD8^+^ T cells in the SVZ of elderly individuals, which was not detected in the dentate gyrus. Moreover, we also found CD3^+^ and CD8^+^ T cells in the SVZ of individuals with neurodegenerative diseases. However, T‐cell count was similar when compared non‐neuropathological elderly with disease diagnosed patients. Our study reveals the infiltration of T cells in old human brains, particularly in the SVZ under non‐pathological conditions and also in neurodegenerative contexts.

## Graphical abstract

Representative image of CD8 in subventricular zone of young and old individual

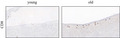

## 1 INTRODUCTION

Age‐related brain impairment promotes a gradual loss of the intrinsic capacity of elderly individuals, which is associated with a progressive cognitive decline that ranges from mild symptoms to severe neurodegenerative diseases such as Alzheimer´s disease (AD), and finishes in death (Bishop, Lu, & Yankner, 2010).

Neurogenesis occurs from neural stem cells (NSCs) in the adult mammalian brain. With age, there is a decline in the number of functional NSCs within neurogenic niches, that is, the subventricular zone (SVZ) of the lateral ventricle and the subgranular zone of the dentate gyrus (DG) in the hippocampus (Capilla‐Gonzalez, Herranz‐Perez, & Garcia‐Verdugo, 2015; Encinas et al., 2011). This correlates with the presence of lower neurogenesis that diminishes the production of new neurons and limits plasticity and repair capacities of the brain, which underlie age‐related cognitive decline (Goncalves, Schafer, & Gage, 2016). NSCs loss or dysfunction also contributes to the onset of age‐related neurodegenerative pathologies (Winner & Winkler, 2015), which are inherently linked with increasing age.

NSC activity is regulated by intrinsic and extrinsic factors (Bond, Ming, & Song, 2015; Fuentealba, Obernier, & Alvarez‐Buylla, 2012). Among the latter, cytotoxic CD8^+^ T‐cell infiltration has been recently detected in the SVZ of the mammalian brain, which impacts NSC proliferation and could contribute to cognitive decline. This effect seems to be dependent on interferon‐γ response (Dulken et al., 2019). Remarkably, CD8^+^ T‐cell infiltration within brain seems to be a stochastic event rather than a passive diffusion due to blood–brain barrier disruption (Dulken et al., 2019; Ritzel et al., 2016). Moreover, once T cells are into the brain neurogenic niches, they may recognize specific antigens and clonally expand to become activated T cells that are different from circulating T cells (Dulken et al., 2019). In this work, we studied the presence of T‐cell infiltration in human neurogenic niches of non‐neuropathological individuals of different ages and also with neurodegenerative diseases.

## 2 RESULTS

### 2.1 CD3^+^ and CD8^+^ T‐cell infiltration is increased in the subventricular zone of aged individuals

**FIGURE 1:**
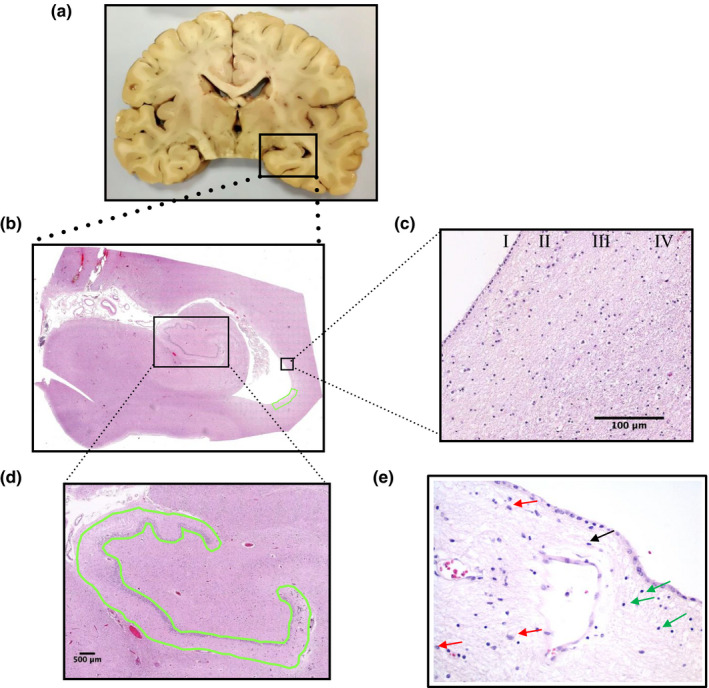
Identification of selected brain regions. (a) Coronal section of adult human brain showing the hippocampus and the temporal horn of the lateral ventricles. (b) Microscopic coronal section showing the hippocampus, entorhinal cortex, and lateral ventricle at the lateral geniculate nucleus level (our reference point). (c) Subventricular zone: We selected an area of 1 mm^2^ that included the four layers (I–IV); all positive cells with immunohistochemical staining that infiltrate the tissue were quantified (excluding those intravascular and vessels wall located). (d) Dentate gyrus: We selected the whole area of this structure, which is defined by three layers (subgranular cell layer, granular cell layer, and molecular layer); all positive cells with immunohistochemical staining that infiltrate the tissue were quantified (excluding those intravascular and vessels wall located). (e) Hematoxylin–eosin staining of the subventricular zone with the main cell types: glial cells (red arrows), T cells (green arrows), and microglia (black arrow)

We investigated the presence of T cells in both neurogenic niches, SVZ and DG, which were obtained in the same section and processed jointly (Figure 1a,b). In the case of SVZ, we defined a surrounding area of 1 mm^2^ (Figure 1c) within which all parenchyma‐infiltrating positive cells, but not those located into blood vessels or vascular walls, were considered. Regarding DG, we encompassed the whole area of this structure (see Figure 1d), excluding again those positive cells located into blood vessels or vascular walls.

The SVZ is a complex niche containing multiple cell types. The most abundant ones are glial cells, which are characterized by a medium, vesicular nucleus with an eosinophilic cytoplasm, sometimes visible, from which fine prolongations project giving them a reticular aspect (Figure 1e; red arrows), and express the GFAP marker (Figure S1). In contrast, microglial cells are characterized by a small, elongated nucleus with a little apparent cytoplasm (Figure 1e; black arrow). The typical morphology of T cells includes a small, round, and dark nucleus with scarce cytoplasm, often very little apparent. Cells with these characteristics were observed in the SVZ of individuals over 65 years old (Figure 1e; green arrows). At the central nervous system (CNS), T cells could be confused with oligodendrocytes, but not in the SVZ, where no myelinated axons are found.

**FIGURE 2:**
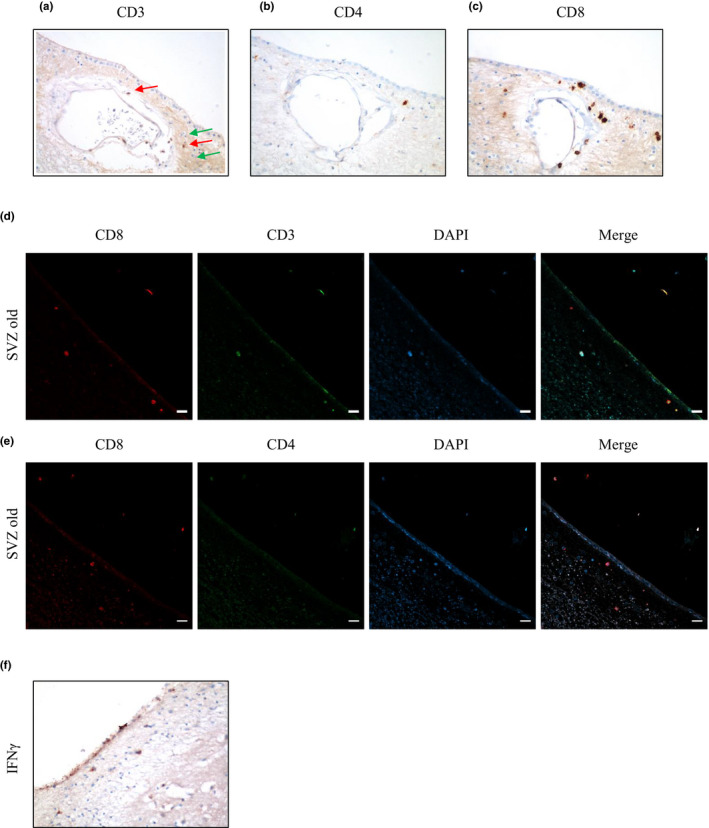
Characterization of CD3‐, CD4‐, and CD8‐positive cells in the subventricular zone of aged individuals. (a) T cells were identified using the CD3 pan T‐cell marker (red arrows). Cells resembling a T‐cell morphology not stained by the CD3 antigen were also observed (green arrows). (b) T helper cells were revealed by CD4 immunostaining. (c) Cytotoxic T cells were identified as positive cells for CD8 antigen. (d) Co‐immunofluorescence of CD8 with CD3 marker. (e) Co‐immunofluorescence of CD8 with CD4 marker. Cell nuclei were counterstained with DAPI. Scale bar: 20 µm. (f) Representative image of interferon‐γ (IFNγ) immunohistochemistry (*n* = 3). Images were obtained using the 40× objective

To characterize the populations of T cells in aged patients, we performed immunohistochemistry (IHC) analyses using the pan T‐cell marker, CD3 (Figure 2a; red arrows). Other cells consistent with a T‐cell morphology not stained using the CD3 antigen were also observed (Figure 2a; green arrows). Moreover, CD4 staining revealed some T helper cells (Figure 2b) and several cells were also co‐stained for CD3 and CD4 markers (Figure S2). IHC further showed the presence of cells expressing the specific marker of cytotoxic T cells, CD8 (Figure 2c). This protein, as well as CD3 and CD4, is membrane protein complexes, so IHC enables to visualize some structures that are not revealed by hematoxylin–eosin, giving cells a larger size than observed after hematoxylin–eosin staining. To further determine the population of cytotoxic T cells, we performed co‐immunofluorescence of CD8 with CD3 or CD4. Confocal analysis revealed that the majority of CD8^+^ cells were also positive for CD3 (Figure 2d) but negative for CD4 markers (Figure 2e). This staining pattern is indicative of the presence of cytotoxic T‐cell infiltration (CD3^+^ CD8^+^ cells) in the SVZ of elderly individuals. Moreover, some cells from SVZ of old individuals expressed high levels of interferon‐γ (Figure 2f).

**FIGURE 3:**
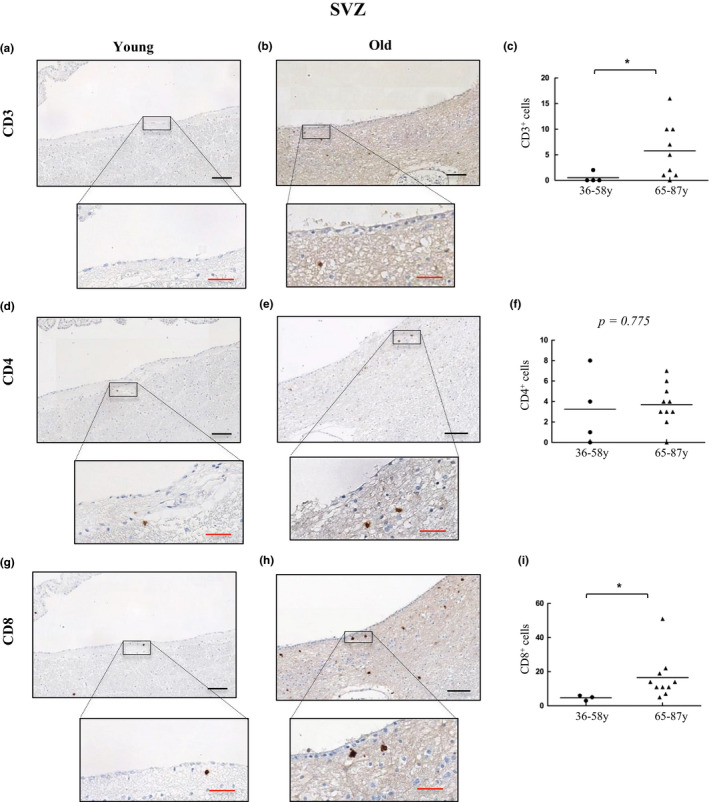
T‐cell infiltration is increased in the subventricular zone with physiological aging. Representative images and quantification of CD3 (a–c), CD4 (d–f), and CD8 (g–i) expression in the subventricular zone of young (*n* = 4) and old individuals (*n* = 10). Data are presented as number of positive cells per mm^2^ with mean bar. Red scale bar: 50 µm; black scale bar: 100 µm

Next, we performed IHC assays to quantify the presence of CD3^+^, CD4^+^, and CD8^+^ cells in samples of SVZ from young and old individuals. CD3^+^ cell quantification ranged from 0 to 2 cells per mm^2^ with only one case showing infiltration in young SVZ (who presented severe liver failure due to herpes viremia), to 0–16 cells per mm^2^ with infiltration in 8 out of 10 individuals and median of 3.5 in old SVZ (Figure 3a–c). CD3^+^ cells were randomly distributed across the different layers of the SVZ. On the other hand, positive cells for CD4 were most frequently found with infiltration in 3 out of 4 young samples with a range of 0–8 cells per mm^2^ (median of 2.5) and 9 out of 10 in old subjects ranging from 0 to 7 cells per mm^2^ (median of 3.5) (Figure 3d–f). CD8^+^ cells infiltrated all SVZ analyzed with a range of 3–6 cells per mm^2^ in young (median of 5) that significantly increases up to 5–51 cells per mm^2^ in old individuals (median of 12.5) (Figure 3g–i). Remarkably, CD8^+^ cells tend to distribute within inner layers (I and II) of SVZ. Our results show that CD3^+^ and CD8^+^ cell subpopulations are increased in elderly individuals, especially in the case of cytotoxic CD8^+^ cells.

**FIGURE 4:**
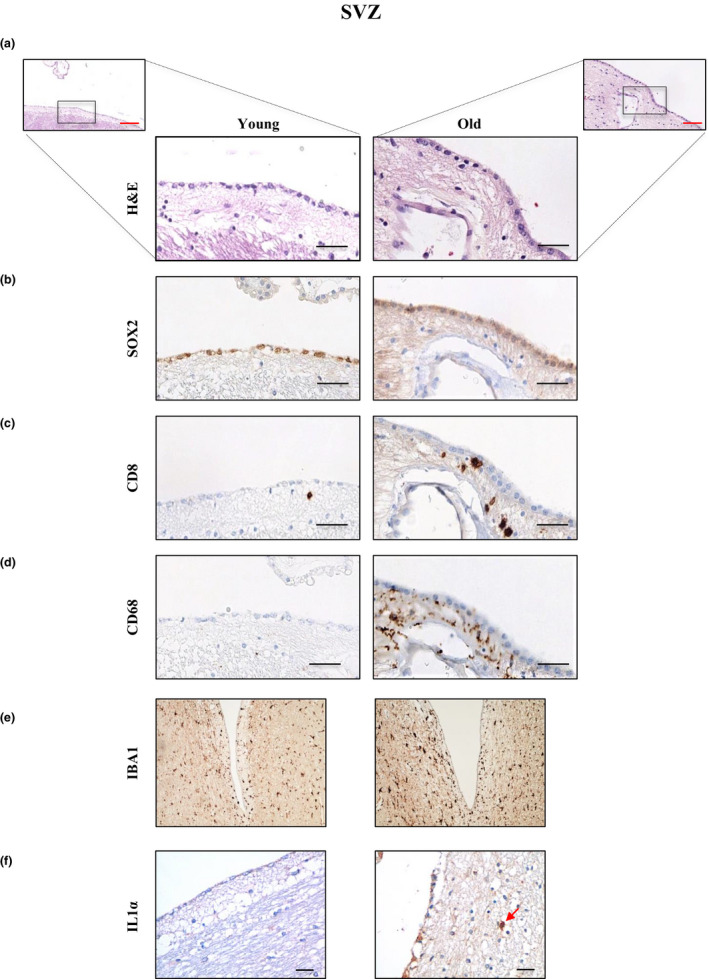
CD8^+^ cells are associated with SOX2, CD68, IBA1, and IL1α expression in the subventricular zone with physiological aging. Representative images of (a) H&E: Hematoxylin–eosin staining, (b) SOX2, (c) CD8, (d) CD68, (e) IBA1, using the 20× objective, and (f) IL1α expression in the subventricular zone of young (*n* = 3) and old individuals (*n* = 7), except for IBA1 and IL1α (*n* = 2 and 3, respectively). Red arrow indicates positive cells for IL1α staining. Red scale bar: 50 µm; black scale bar: 100 µm

To check the link between the population of infiltrating CD8^+^ cells with NSCs, we studied the expression of SOX2 as NSC marker. IHC assays revealed a reduced expression of SOX2 in samples from old individuals (Figure 4a,b). Interestingly, we observed the close proximity of CD8^+^ cells to those cells positive for SOX2 in old SVZ (Figure 4b,c). These data indicate that cytotoxic CD8^+^ cells localize in the vicinity of NSCs in the SVZ. In contrast, we did not detect a clear correlation between the expression of CD8 and p16^INK4a^, marker of senescence, although p16^INK4a^ staining was more elevated with age (Figure S3). On the other hand, microglial cells are the immune effector cells of the CNS and derive from the monocyte–macrophage lineage, so they share common IHC markers with peripheral macrophages such as CD68. Thus, we determined the expression of CD68 as well as IBA1, an additional marker of microglia. IHC staining revealed the elevated expression of both of them in samples from old individuals, which also presented morphological changes such as enlargement of their soma and shortening of their cellular processes indicative of activated microglia (Figure 4c–e). Moreover, there was a positive correlation between CD68 and CD8 expression, since CD68 was more intensely stained in those samples with higher levels of CD8^+^ cells (Figure 4c,d). Finally, we performed IHC of the pro‐inflammatory cytokine IL1α and found that its levels were more elevated in elderly individuals (Figure 4f). Our results indicate that infiltrating cytotoxic CD8^+^ cells are associated with a pro‐inflammatory context in aged individuals.

### 2.2 CD3^+^ and CD8^+^ T cells do not infiltrate the dentate gyrus in aged individuals

**FIGURE 5:**
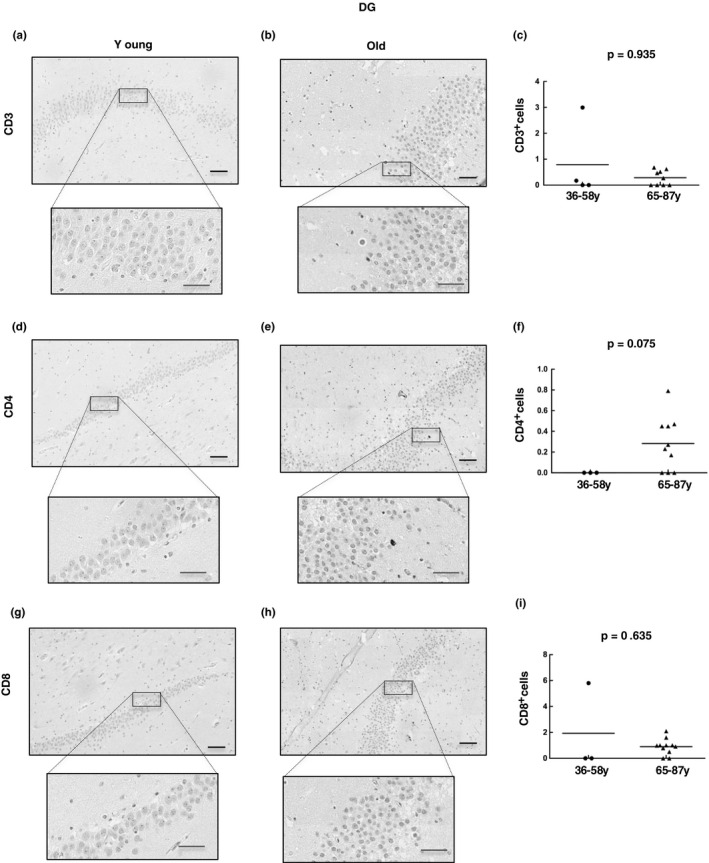
T‐cell infiltration is very low in the dentate gyrus with physiological aging. Representative images and quantification of CD3 (a–c), CD4 (d–f), and CD8 (g–i) expression in the dentate gyrus of young (*n* = 4) and old individuals (*n* = 10). Data are presented as number of positive cells per mm^2^ with mean bar. Red scale bar: 50 µm; black scale bar: 100 µm

Next, we moved to the other neurogenic niche in adults, that is, the DG, and performed the same analyses of CD3, CD4, and CD8 expression by IHC. In the young group, we observed a count of CD3^+^ T cells less than 1 in all cases except for one patient with 3 cells per mm^2^ who presented severe liver failure due to herpes viremia (Figure 5a–c); aged patients presented similar values with less than 1 cell per mm^2^ in all cases. CD4 expression followed this pattern in both groups (Figure 5d–f), and the same results were obtained for the CD8 marker (with less than 1 cell positive per mm^2^ in the young group and 0–2.1 positive cells per mm^2^ in the old group) (Figure 5g–i), always excluding the patient with herpes viremia abovementioned. These results point out that T‐cell infiltration in the DG is not increased with aging unlike that observed in the SVZ.

### 2.3 CD3^+^ and CD8^+^ T cells are present in subventricular zone of aged individuals with neurodegenerative diseases

**FIGURE 6:**
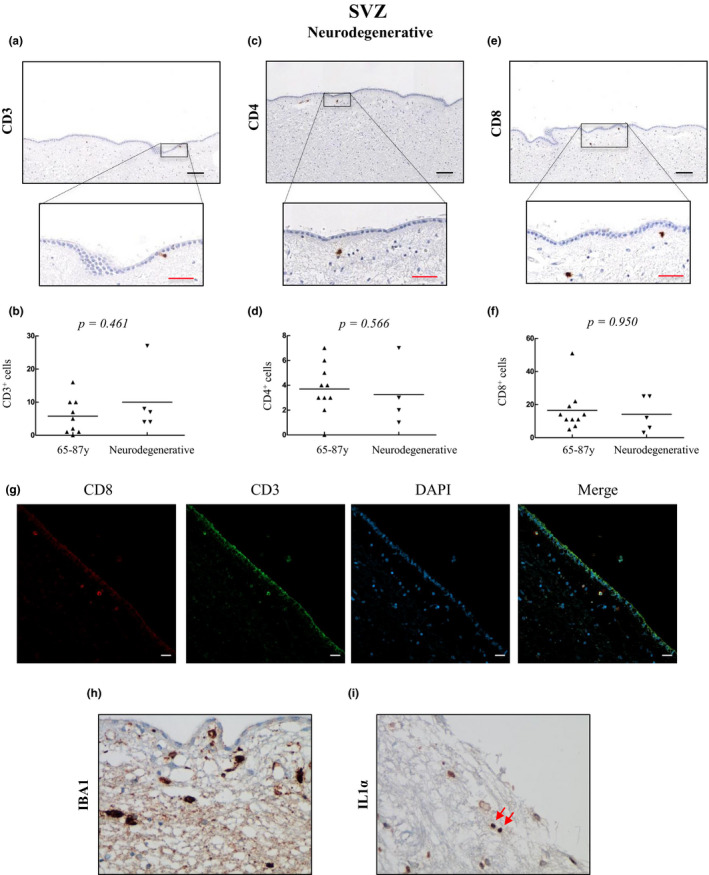
T‐cell infiltration is increased in the subventricular zone with pathological aging. Representative images and quantification of CD3 (a, b), CD4 (c, d), and CD8 (e, f) expression in the subventricular zone of patients with neurodegenerative diseases (*n* = 5) and comparison with numbers detected in old individuals. Data are presented as number of positive cells per mm^2^ with mean bar. Red scale bar: 50 µm; black scale bar: 100 µm. (g) Co‐immunofluorescence of CD8 with CD3 marker in the subventricular zone of patients with neurodegenerative diseases (*n* = 2); scale bar: 20 µm. (h–i) Representative images of IBA1 and IL1α staining were obtained using the 40× objective (*n* = 2). Red arrows indicate positive cells for IL1α staining

We finally wondered whether T‐cell infiltration could impact patients with neurodegenerative diseases. For that purpose, we analyzed the presence of CD3^+^, CD4^+^, and CD8^+^ cells in individuals diagnosed with AD. Regarding T‐cell counts in the SVZ, CD3^+^ cells were present in all patients with a range of 4–27 cells per mm^2^ (median of 7) in comparison with 0–16 cells per mm^2^ (median of 3.5) in aged patients with no neuropathological signs (Figure 6a,b). CD4^+^ T cells were detected in all patients with neurodegeneration (1–7 cells per mm^2^; median of 3), maintaining comparable levels to the old group (0–7 cells per mm^2^; median of 3.5) (Figure 6c,d). CD8^+^ cells were also found in all patients (3–25 cells per mm^2^; median of 12) at similar levels as for the old group 5–51 cells per mm^2^ (median of 12.5) (Figure 6e,f). Confocal analysis showed that the majority of CD8^+^ cells were positive for CD3 (Figure 6g). Additionally, SVZ samples from those patients also presented intense staining for IBA1 (Figure 6h) and positive cells for IL1α (Figure 6i). Our data show that T‐cell infiltration occurs in the SVZ of neurodegenerative patients but at similar levels as found in aged brains without neuropathological injury.

**FIGURE 7:**
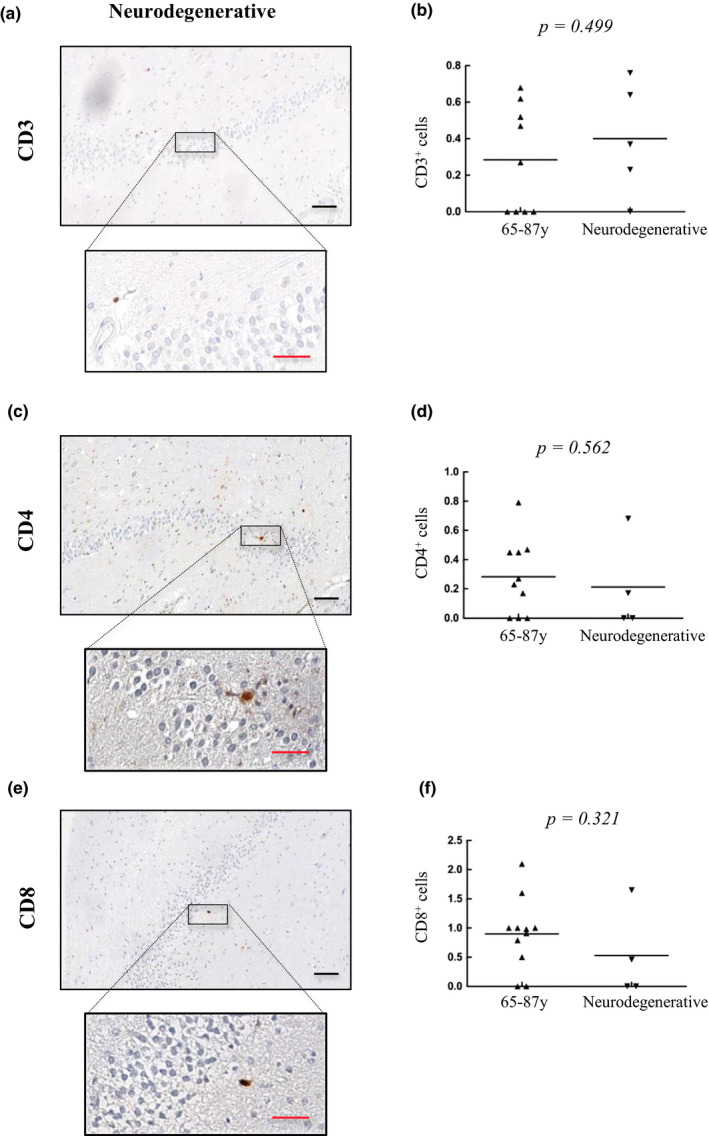
T‐cell infiltration is very low in the dentate gyrus with pathological aging. Representative images and quantification of CD3 (a, b), CD4 (c, d), and CD8 (e, f) expression in the dentate gyrus of patients with neurodegenerative diseases (*n* = 5) and comparison with numbers detected in old individuals. Data are presented as number of positive cells per mm^2^ with mean bar. Red scale bar: 50 µm; black scale bar: 100 µm

As in the case of the elderly group, very low number of CD3^+^ (Figure 7a,b), CD4^+^ (Figure 7c,d), and CD8^+^ cells (Figure 7e,f), and no differences between groups, were detected in the DG of patients with neurodegenerative diseases. These results confirm that T‐cell infiltration in the DG is anecdotal during physiological and pathological aging.

## 3 DISCUSSION

In this study, we provide new evidence about the existence of T‐cell infiltration in brain with physiological and pathological aging. A recent report by Brunet´s Lab showed that T‐cell infiltration occurs in old mice and human SVZ, where CD3^+^ and CD8^+^ cells were elevated when compared to young individuals (Dulken et al., 2019). In our work, we extend these data and show that CD3^+^ and CD8^+^ cells are increased in the SVZ of elderly individuals but not in the DG, and they are also present in neurodegenerative diseases such as AD. We found higher SVZ CD8^+^ total counts than Dulken´s study although the ratio of increase between young and old individuals was the same in both works. This could be due to different inclusion criteria of patients (Table 1), different antibodies employed or the anatomical region considered, since they studied the SVZ at basal ganglia level and we did so at temporal horn. Concerning this, it is important to mention that we selected this region to obtain both neurogenic niches in the same section in order to be processed jointly. Conversely, our results show that T‐cell infiltration is negligible in the DG of both elderly individuals and patients with neurodegenerative diseases unlike we observed in the SVZ, which points to a specific role of T‐cell infiltration in the SVZ with aging. Intriguingly, we detected a higher proportion of CD8^+^ cells than CD3^+^ cells in human SVZ, which might suggest the presence of at least one CD8^+^ cell subpopulation different from cytotoxic CD3^+^ CD8^+^ T cells. In this sense, some natural killer (NK) cells carry CD8 but not the CD3 pan T‐cell marker (Campbell, Guy, Cosgrove, Florida‐James, & Simpson, 2008) and also subsets of CD8^+^ cells with markers of NK cells have been identified in human peripheral blood (Ohkawa et al., 2001).

**TABLE 1:** Relevant clinical information of young and aged individuals included in this study

Group	Age	Gender	Diagnosis
Young	43	M	Severe acute liver failure and herpes viremia
	36	F	Toxic shock
	47	M	Neuroendocrine carcinoma and encephalopathy
	58	M	Disseminated signet ring cell carcinoma
Old	66	M	Disseminated breast carcinoma
	67	M	Laryngeal squamous cell carcinoma and respiratory depression
	65	M	Pulmonary hemorrhage
	77	M	Septic shock
	74	F	Disseminated breast carcinoma
	85	M	Malignant mesothelioma
	78	F	Hypovolemic shock
	70	F	Metastatic melanoma
	85	M	Subdural hematoma and sudden death
	87	M	Empyema

It is also important to mention that we described the same trend of T‐cell infiltration in neurodegenerative diseases as we found in aged individuals with no neuropathological injuries (presence of CD3^+^ and CD8^+^ T cells in the SVZ but not in the DG). All our patients with a neurodegenerative disease displayed AD. The contribution of brain inflammation and T cells to AD is still controversial, but other groups have also found CD8^+^ cells in the brain parenchyma from patients with AD (Itagaki, McGeer, & Akiyama, 1988; Liang et al., 2017; Rogers, Luber‐Narod, Styren, & Civin, 1988). Although we cannot infer anything about if the presence of T cells is contributing to the neurodegenerative disease of these patients, our results suggest that accumulation of CD8^+^ cells is not associated with progression in neural degeneration from non‐pathological to pathological conditions.

Finally, we observed a distribution trend of CD8^+^ T cells in the superficial layers of the SVZ, particularly in the vicinity of SOX2^+^ NSCs in the ependymal layer. This, together with the reduction in SOX2 expression with age, the presence of cells with intense staining for interferon‐γ, and the evidence that CD8^+^ T cells inhibit NSC proliferation (Dulken et al., 2019), might indicate that CD8^+^ cells infiltrating the human SVZ trigger a cytotoxic immune response in this neurogenic niche, eventually leading to a reduction in the NSC pool in aged individuals. The increased expression of CD68 and IBA1 microglia markers as well as IL1α in the aged SVZ, which suggest a pro‐inflammatory environment into the niche, may support this point.

In conclusion, we report an increase in CD8^+^ T cells in aged human SVZ, but not in the DG, which is also observed in neurodegenerative diseases at the same level. Our results point out to a specific role of inflammation in human SVZ associated with physiological and pathological aging.

## 4 MATERIAL AND METHODS

### 4.1 Samples

Human brain samples were collected from autopsies conducted at Donostia University Hospital (Spain). Postmortem interval (PMI) was limited to 12 hr due to its effects on brain proteins. Brains were kept in a fixative solution (4% paraformaldehyde) for a period of not less than 24 hr. Samples were divided into three groups: “young” (*n* = 4; individuals ranged from 36 to 58 years old), “old” (*n* = 10; individuals aged 65‐87 years old), and “neurodegenerative” (*n* = 5; individuals between 60 and 90 years old). Inclusion criteria for selection of “young” and “old” patients included the absence of diagnosis of neurodegenerative disorders as well as the lack of neuropathological injuries in the regions analyzed. Clinical data are available in Tables 1 and 2.

**TABLE 2:** Relevant clinical information of patients with neurodegenerative diseases

Age	Gender	Diagnosis
90	F	‐ Alzheimer´s disease ‐ Stage vi BRAAK (tauopathy) ‐ Stage 5 of THAL (amyloid deposits)
79	M	‐ Alzheimer´s disease ‐ Stage i BRAAK ‐ Stage 5 of THAL
71	M	‐ Alzheimer´s disease ‐ Stage i BRAAK ‐ Stage 5 of THAL
60	F	‐ Alzheimer´s disease ‐ Stage vi BRAAK ‐ Stage 5 of THAL
65	F	‐ Alzheimer´s disease ‐ Stage vi BRAAK ‐ Stage 5 of THAL

This study was approved by the Clinical Research Ethics Committee of the Donostia University Hospital (AMM‐MHP‐2019‐1) and adhered to the tenets of the Declaration of Helsinki by the World Medical Association regarding human experimentation.

### 4.2 Immunohistochemistry of brain sections

IHC was performed following standard procedures. Briefly, whole brains were extracted, fixed in formalin, paraffin‐embedded, and subsequently sectioned in 5 µm coronal sections with a Leica rotary microtome. Sections were taken at lateral geniculate nucleus of the thalamus in the temporal lobe, which was considered our topographic reference point. All sections included hippocampus, temporal horn, and entorhinal cortex. The following primary antibodies were used: CD3 (Roche, Ref.: 790‐4341, Clone: 2GV6), CD4 (Roche, Ref.: 790‐4423, Clone: SP35), CD8 (Roche, Ref.: 790‐4460, Clone: SP57), CD68 (Roche, Ref.: 790‐2931, Clone: KP‐1), IBA1 (Wako; Ref.: 019‐19741), GFAP (Roche, Ref.: 760‐4345), Interferon‐γ (Abcam, Ref.: ab9657), IL1α (Abcam, Ref.: ab9614), SOX2 (Cell Marque™, Ref.: 760‐4621, Clone: SP76), and p16^INK4a^ (Roche, Ref.: 805‐4713). IHC was performed following the manufacturer´s instructions on the *Roche Ventana Benchmark ULTRA System* with ethylenediaminetetraacetic acid (EDTA) pH 8.5 antigen retrieval. Hematoxylin–eosin staining of SVZ sections was performed using standard procedures. Sections were visualized with a light microscope using the 10×, 20×, and 40× objective and then scanned with *Virtuoso* v.5.6.1 software (Ventana Medical Systems, Roche).

### 4.3 T‐cell quantification

The quantification of positive T cells for the different markers in the SVZ and the entire DG was manually performed in entire coronal sections from previously scanned images (Figure 1a,b). In the case of SVZ, we defined a surrounding area of 1 mm^2^ (Figure 1c). All parenchyma‐infiltrating positive cells within this area, but not those located into blood vessels or vascular walls, were counted and considered for further analyses. Regarding DG, we encompassed the whole area of this structure (see Figure 1d), counted the total number of positive cells in such area, excluding those located into blood vessels or vascular walls, and normalized this number to the area considered. T cells were identified as CD3^+^ cells, helper T cells as CD4^+^, and cytotoxic T cells as CD8^+^.

### 4.4 Immunofluorescence of brain sections

Immunofluorescence was performed in formalin‐fixed brain samples. Paraffin‐embedded tissue sections were deparaffinized in xylene and rehydrated in a series of graded alcohols and then heated in citrate buffer for 30 min for antigen retrieval. Tissues were permeabilized with 0.5% Triton X‐100 (PBS‐T; T8787, Sigma‐Aldrich) and blocked for 1 hr with 1% bovine serum albumin and 5% goat serum (G9023, Sigma‐Aldrich) in PBS‐T. Sections were incubated at 4ºC overnight with the following primary antibodies: anti‐CD3 (ab5690, Abcam), CD4 (ab133616, Abcam), and CD8 (ab4055, Abcam). The sections were washed 3 times for 5 min with PBS 0.1% Tween‐20 (822184, Sigma‐Aldrich) and incubated for 1 hr at room temperature in darkness with Alexa Fluor 488 goat anti‐rabbit (A32731, Invitrogen) and Alexa Fluor 555 goat anti‐rabbit (A32732, Invitrogen) secondary antibodies. Nuclear DNA was stained with DAPI (D9542, Sigma‐Aldrich). The preparation was mounted with Fluoro‐Gel mounting media (17985‐10, Aname), and immunofluorescence was evaluated with the Zeiss LSM 900 confocal microscope.

### 4.5 Statistical analysis

The number of T cells was expressed as total number of positive cells per unit area (mm^2^). Two‐tailed Mann–Whitney *U* test was performed to compare CD3‐, CD4‐, and CD8‐positive cell counts between groups. Asterisks (*, **, and ***) indicate statistically significant differences (*p* < 0.05, *p* < 0.01, and *p* < 0.001, respectively).

## CONFLICT OF INTEREST

None.

## AUTHOR CONTRIBUTIONS

M.M‐V, TM.S, and JP.G performed the autopsies, selected the samples and biopsies, performed immunohistochemistry, and took pictures. A.S‐A performed the immunofluorescence studies. L.M‐C and M.A‐S analyzed the results, quantified the data, and elaborated the figures. M.M‐V and M.A‐S helped in the writing of the manuscript. All of them revised the manuscript. A.M. designed the research, directed the project, obtained funds, and wrote the manuscript.

## Supporting information

Figures S1‐S3

## Data Availability

The data that support the findings of this study are available from the corresponding author upon reasonable request.

## References

[acel13198-bib-0001] Bishop, N. A. , Lu, T. , & Yankner, B. A. (2010). Neural mechanisms of ageing and cognitive decline. Nature, 464, 529–535. 20336135 10.1038/nature08983 PMC2927852

[acel13198-bib-0002] Bond, A. M. , Ming, G. L. , & Song, H. (2015). Adult mammalian neural stem cells and neurogenesis: Five decades later. Cell Stem Cell, 17, 385–395. 26431181 10.1016/j.stem.2015.09.003 PMC4683085

[acel13198-bib-0003] Campbell, J. P. , Guy, K. , Cosgrove, C. , Florida‐James, G. D. , & Simpson, R. J. (2008). Total lymphocyte CD8 expression is not a reliable marker of cytotoxic T‐cell populations in human peripheral blood following an acute bout of high‐intensity exercise. Brain, Behavior, and Immunity, 22, 375–380. 10.1016/j.bbi.2007.09.001 17949944

[acel13198-bib-0004] Capilla‐Gonzalez, V. , Herranz‐Perez, V. , & Garcia‐Verdugo, J. M. (2015). The aged brain: Genesis and fate of residual progenitor cells in the subventricular zone. Front Cell Neurosci., 9, 365. 26441536 10.3389/fncel.2015.00365 PMC4585225

[acel13198-bib-0005] Dulken, B. W. , Buckley, M. T. , Navarro Negredo, P. , Saligrama, N. , Cayrol, R. , Leeman, D. S. , … Brunet, A. (2019). Single‐cell analysis reveals T cell infiltration in old neurogenic niches. Nature, 571, 205–210. 31270459 10.1038/s41586-019-1362-5 PMC7111535

[acel13198-bib-0006] Encinas, J. M. , Michurina, T. V. , Peunova, N. , Park, J. H. , Tordo, J. , Peterson, D. A. , … Enikolopov, G. (2011). Division‐coupled astrocytic differentiation and age‐related depletion of neural stem cells in the adult hippocampus. Cell Stem Cell, 8, 566–579. 21549330 10.1016/j.stem.2011.03.010 PMC3286186

[acel13198-bib-0007] Fuentealba, L. C. , Obernier, K. , & Alvarez‐Buylla, A. (2012). Adult neural stem cells bridge their niche. Cell Stem Cell, 10, 698–708. 22704510 10.1016/j.stem.2012.05.012 PMC3726005

[acel13198-bib-0008] Goncalves, J. T. , Schafer, S. T. , & Gage, F. H. (2016). Adult neurogenesis in the hippocampus: From stem cells to behavior. Cell, 167, 897–914. 27814520 10.1016/j.cell.2016.10.021

[acel13198-bib-0009] Itagaki, S. , McGeer, P. L. , & Akiyama, H. (1988). Presence of T‐cytotoxic suppressor and leucocyte common antigen positive cells in Alzheimer's disease brain tissue. Neuroscience Letters, 91, 259–264. 2972943 10.1016/0304-3940(88)90690-8

[acel13198-bib-0010] Liang, Z. , Zhao, Y. , Ruan, L. , Zhu, L. , Jin, K. , Zhuge, Q. , … Zhao, Y. (2017). Impact of aging immune system on neurodegeneration and potential immunotherapies. Progress in Neurobiology, 157, 2–28. 28782588 10.1016/j.pneurobio.2017.07.006

[acel13198-bib-0011] Ohkawa, T. , Seki, S. , Dobashi, H. , Koike, Y. , Habu, Y. , Ami, K. , … Sekine, I. (2001). Systematic characterization of human CD8+ T cells with natural killer cell markers in comparison with natural killer cells and normal CD8+ T cells. Immunology, 103, 281–290. 11454057 10.1046/j.1365-2567.2001.01248.x PMC1783250

[acel13198-bib-0012] Ritzel, R. M. , Crapser, J. , Patel, A. R. , Verma, R. , Grenier, J. M. , Chauhan, A. , … McCullough, L. D. (2016). Age‐associated resident memory CD8 T cells in the central nervous system are primed to potentiate inflammation after ischemic brain injury. The Journal of Immunology, 196, 3318–3330. 26962232 10.4049/jimmunol.1502021 PMC4868658

[acel13198-bib-0013] Rogers, J. , Luber‐Narod, J. , Styren, S. D. , & Civin, W. H. (1988). Expression of immune system‐associated antigens by cells of the human central nervous system: relationship to the pathology of Alzheimer's disease. Neurobiology of Aging, 9, 339–349. 3263583 10.1016/s0197-4580(88)80079-4

[acel13198-bib-0014] Winner, B. , & Winkler, J. (2015). Adult neurogenesis in neurodegenerative diseases. Cold Spring Harb Perspect Biol., 7, a021287. 25833845 10.1101/cshperspect.a021287 PMC4382734

