# Supplementary material for: CD8+ T cells are increased in the subventricular zone with physiological and pathological aging
Source: Aging Cell. 2020 Aug 1;19(9):e13198. doi: 10.1111/acel.13198 (PMC7511866; doi:10.1111/acel.13198)
Supplement: Supplementary file 1 — Figures S1‐S3 [file ACEL-19-e13198-s001.pptx]

## Slide 1
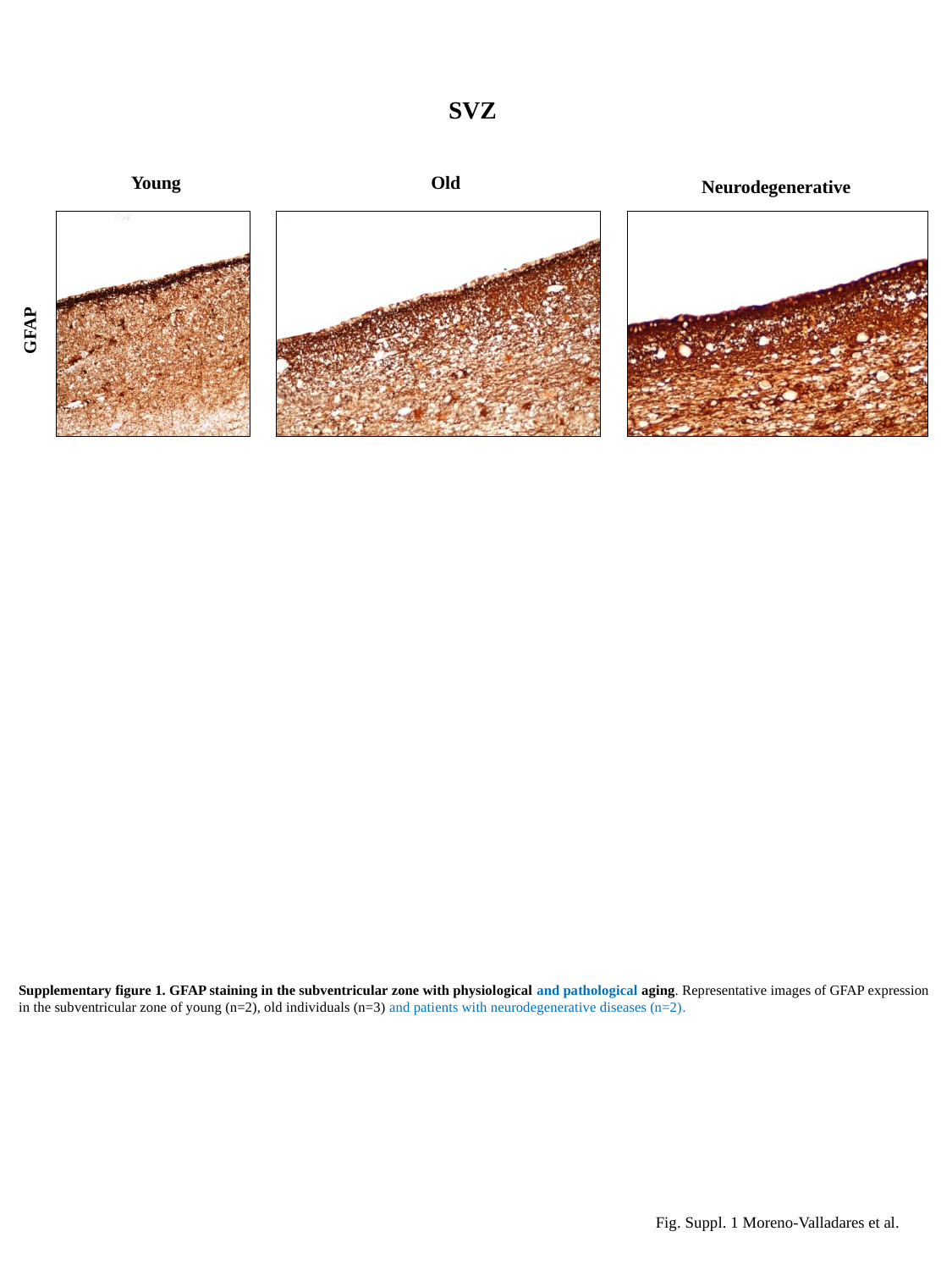

SVZ
Young
Old
Neurodegenerative
GFAP
Supplementary figure 1. GFAP staining in the subventricular zone with physiological and pathological aging. Representative images of GFAP expression in the subventricular zone of young (n=2), old individuals (n=3) and patients with neurodegenerative diseases (n=2).
Fig. Suppl. 1 Moreno-Valladares et al.

## Slide 2
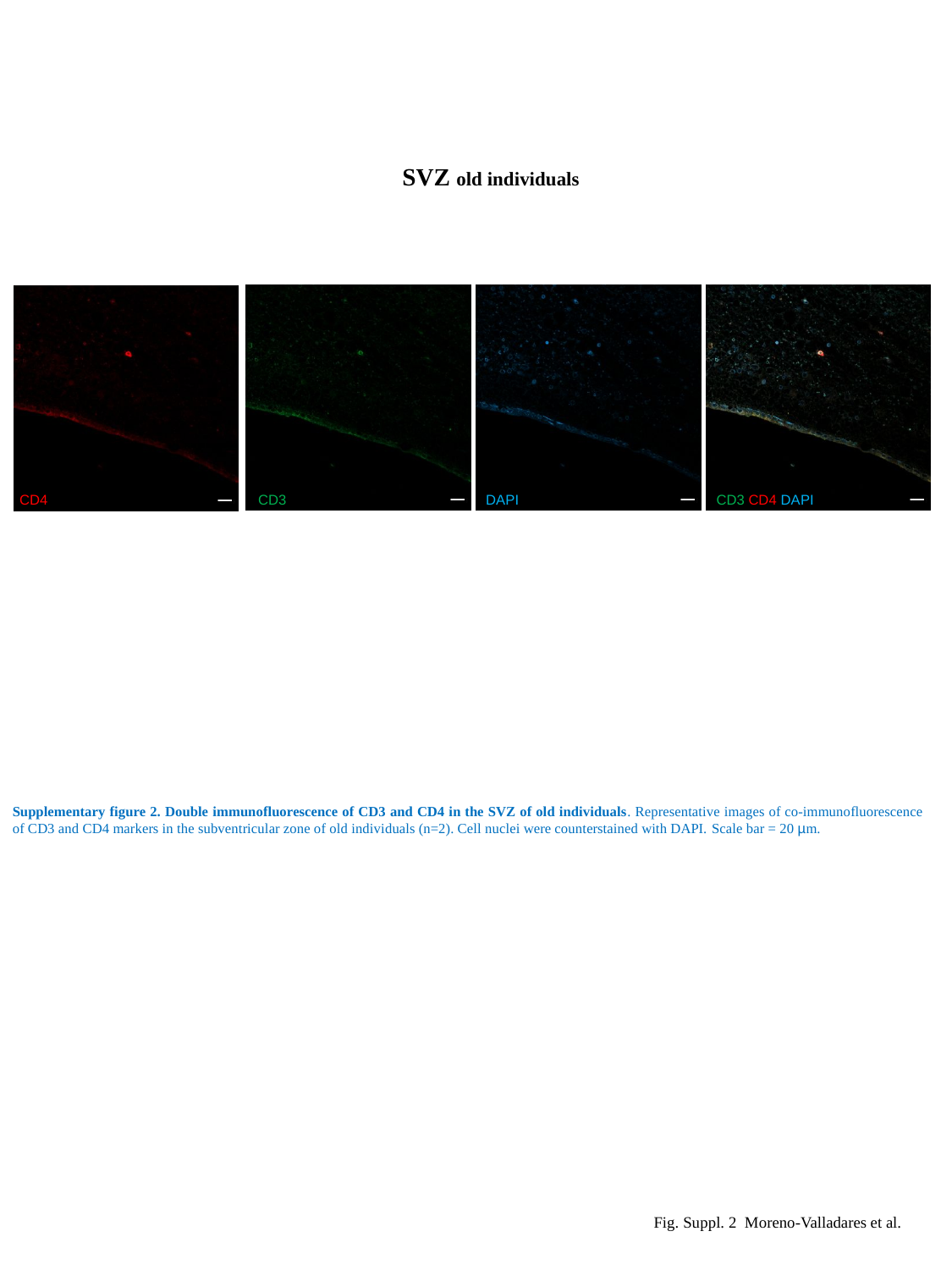

SVZ old individuals
CD4
CD3
DAPI
CD3 CD4 DAPI
Supplementary figure 2. Double immunofluorescence of CD3 and CD4 in the SVZ of old individuals. Representative images of co-immunofluorescence of CD3 and CD4 markers in the subventricular zone of old individuals (n=2). Cell nuclei were counterstained with DAPI. Scale bar = 20 µm.
Fig. Suppl. 2 Moreno-Valladares et al.

## Slide 3
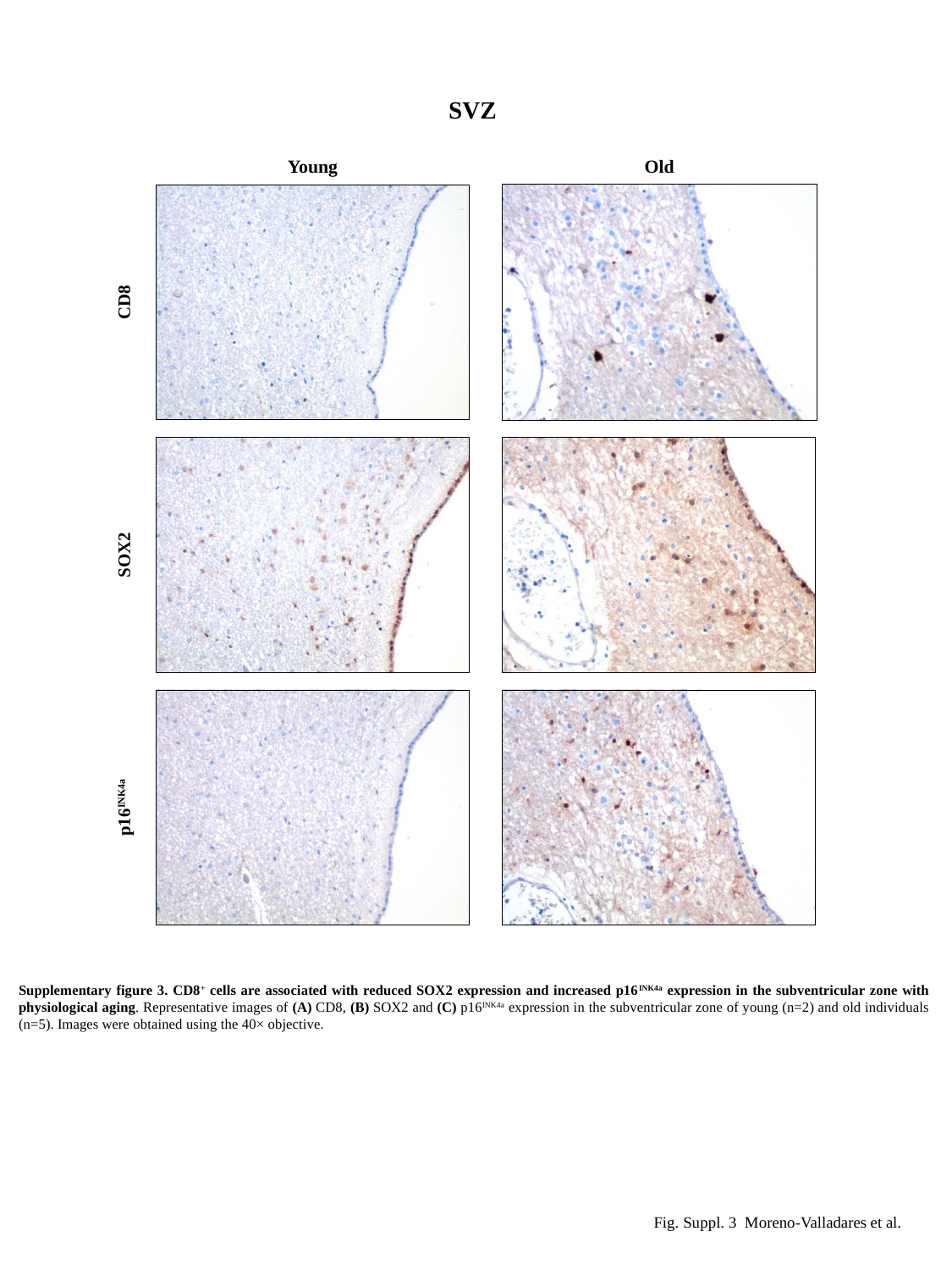

SVZ
Young
Old
CD8
SOX2
p16INK4a
Supplementary figure 3. CD8+ cells are associated with reduced SOX2 expression and increased p16INK4a expression in the subventricular zone with physiological aging. Representative images of (A) CD8, (B) SOX2 and (C) p16INK4a expression in the subventricular zone of young (n=2) and old individuals (n=5). Images were obtained using the 40× objective.
Fig. Suppl. 3 Moreno-Valladares et al.
